# Identification of the Type IX Secretion System Component, PorV (CHU_3238), Involved in Secretion and Localization of Proteins in *Cytophaga hutchinsonii*

**DOI:** 10.3389/fmicb.2021.742673

**Published:** 2021-10-20

**Authors:** Dong Zhao, Wenxia Song, Sen Wang, Weican Zhang, Yue Zhao, Xuemei Lu

**Affiliations:** ^1^State Key Laboratory of Microbial Technology, Shandong University, Qingdao, China; ^2^School of Medicine, Cheeloo College of Medicine, Shandong University, Jinan, China; ^3^Qingdao Institute of Bioenergy and Bioprocess Technology, Chinese Academy of Sciences, Qingdao, China

**Keywords:** *Cytophaga hutchinsonii*, type IX secretion system, PorV, cellulose degradation, gliding motility, protein secretion

## Abstract

*Cytophaga hutchinsonii* can efficiently degrade cellulose and rapidly glide over surfaces, but the underlying mechanisms remain unclear. The type IX secretion system (T9SS) is involved in protein secretion and gliding motility, which is unique to the phylum *Bacteroidetes*. In this study, we deleted a homologous gene of *PorV* (*chu_3238*), a shuttle protein in the T9SS. The Δ3238 mutant caused cellulolytic and gliding defects, while the *porV* deletion mutants in other *Bacteroidetes* could glide normally. Adding Ca^2+^ and K^+^ improved growth in the PY6 medium, suggesting a potential role of *chu_3238* in ion uptake. A proteomic analysis showed an increase in the number of extracellular proteins in the Δ3238 mutant and a decrease in the outer membrane proteins compared to the wild type (WT). Endoglucanase activity in the Δ3238 intact cells was reduced by approximately 70% compared to that of the WT. These results indicate that the secreted proteins could not attach to the cell surface but were released into the extracellular space in the Δ3238 mutant. However, the cargo proteins accumulated in the periplasm of other reported *porV* deletion mutants. In addition, the homologs of the translocon SprA and a Plug protein were pulled down by co-immunoprecipitation in the 3238-FLAG strain, which are involved in protein transport in the T9SS of *Flavobacterium johnsoniae*. The integrity of the lipopolysaccharide (LPS) was also affected in the Δ3238 mutant, which may be the reason for the sensitivity of the cell to toxic reagents. The functional diversity of CHU_3238 suggests its important role in the T9SS of *C. hutchinsonii* and highlights the functional differences of PorV in the T9SS among the *Bacteroidetes*.

## Introduction

The type IX secretion system (T9SS), which consists of a subset of gliding proteins, is common and unique to the phylum *Bacteroidetes* (McBride and Zhu, 2013). The T9SS of this phylum secretes various functional proteins related to gliding motility, chitin utilization in *Flavobacterium johnsoniae* (Sato et al., 2010; Rhodes et al., 2011; McBride and Zhu, 2013), and gingipain-associated virulence factors in *Porphyromonas gingivalis* (Benedyk et al., 2019; Veillard et al., 2019). The T9SS also plays a role in bacterial diseases in birds and fish, including rainbow trout fry syndrome and cold-water disease caused by *Bacteroidetes* (Guo et al., 2017; Li et al., 2017; Perez-Pascual et al., 2017; Barbier et al., 2020). At least 21 protein components are found in the T9SS, and although interacting partners of many T9SS proteins are known, how the sub-complexes are associating is a mystery (Lasica et al., 2017; Gorasia et al., 2020a, b). The N-terminal signal peptides direct the substrate proteins of the T9SS to cross the cytoplasmic membrane through the Sec system, while the conserved C-terminal domains (CTDs) that are unique to the T9SS guide substrates across the outer membrane (McBride and Zhu, 2013; Veith et al., 2013; Gorasia et al., 2020a, b). CTDs are classified into two types: type A and type B (McBride and Zhu, 2013). The T9SS is also responsible for the modification and position of substrate proteins (Gorasia et al., 2015). PorV is the most abundant T9SS component (Gorasia et al., 2020a), which may be related to its multiple functions in the T9SS. As an outer membrane β-barrel shuttle protein, PorV forms a complex with the translocon SprA/Sov to accept cargo proteins that are transported by the periplasmic T9SS complex component to the cell surface (Lauber et al., 2018; Gorasia et al., 2020b). The cargo proteins are delivered and their CTDs are cleaved by the C-terminal signal peptidase (PorU), which is localized to the outer membrane through interaction with PorV (Glew et al., 2017). The proteins are secreted into the extracellular matrix or covalently link to an anionic lipopolysaccharide (A-LPS), which requires PorV for *O*-deacylation of the outer membrane (Miot and Betton, 2004; Chen et al., 2011; Gorasia et al., 2015; Kharade and McBride, 2015; Glew et al., 2017). In this way, the T9SS finishes the transportation and localization of the CTD proteins.

*Cytophaga hutchinsonii*, which belongs to the phylum *Bacteroidetes*, is a Gram-negative aerobic soil bacterium. It can efficiently and rapidly digest insoluble crystalline cellulose and exhibits gliding motility over surfaces (Walker and Warren, 1938; Stanier, 1942; Bayer et al., 2004). Two strategies for microbial cellulose degradation are well known: many aerobic microorganisms adopt a free-cellulase mechanism, whereas most anaerobic microorganisms utilize multienzyme cellulosomes (Bayer et al., 2004; Zhang and Lynd, 2004; Wilson, 2008). However, *C. hutchinsonii* appears to adopt a novel cellulose-degradation mechanism, which is different from the two strategies described above. As most of the cellulases need to associate with cells to exhibit their activity, it is crucial for *C. hutchinsonii* to interact directly with cellulose when degrading it (Ji et al., 2014). However, the cellulose-binding mechanism of *C. hutchinsonii* is unclear because it does not have cellulose-binding modules (CBMs) or type IV pili capable of binding to cellulose (Zhu and McBride, 2017). *C. hutchinsonii* expresses all the orthologs of the T9SS genes and has 147 proteins with predicted CTDs including 12 endoglucanases, some of which have been found on the cell surface (Veith et al., 2013; Kulkarni et al., 2017; Zhu and McBride, 2017). Moreover, outer membrane proteins are necessary for *C. hutchinsonii* to bind and degrade cellulose, and many of these proteins are the components or substrates of the T9SS (Ji et al., 2012, 2014; Wang et al., 2017, 2019; Zhao et al., 2020). The components of *C. hutchinsonii*, including CHU_0170 (SprP), CHU_3237 (PorU), CHU_0174 (GldN), CHU_0029 (SprA), and CHU_2709 (SprT), were found to affect the gliding motility and cellulose utilization (Wang et al., 2014; Zhu and McBride, 2014; Gao et al., 2020; Gao et al., 2021). In addition, CHU_0174, CHU_0029, and CHU_2709 were found to be essential for the assimilation of Ca^2+^ and Mg^2+^ (Gao et al., 2020, 2021). Ion channels are required for most nutrients’ passive transportation into the periplasm, which is beneficial for bacterial growth. A previous study reported that a T6SS from *Pseudomonas* secreted a protein that could cooperate with the Fe(III)-pyochelin receptor, FptA, and the porin OprF to transfer iron from outer membrane vesicles to bacterial cells (Lin et al., 2017). However, the mechanism by which T9SS is involved in ion uptake is unknown.

The aim of this study is to reveal the functions of components of the T9SS in order to understand their role in *C. hutchinsonii*. CHU_3238, which is located upstream of CHU_3237 (PorU), showed 38.6% amino sequence identity with PorV. Deletion of the *chu_3238* in *C. hutchinsonii* caused growth defects in PY6 medium, as well as cellulolytic and colony spreading defects. An analysis of the phenotypes of the *chu_3238* deletion mutant revealed its multiple roles in the T9SS.

## Materials and Methods

### Bacterial Strains, Plasmids, and Culture Conditions

Bacterial strains and plasmids are listed in Table 1, and primers used for amplification are listed in Table 2. PY6 medium (6 g/l peptone and 0.5 g/l yeast extract) with 4 g/l glucose, Stanier medium (1 g/l KNO_3_, 1 g/l K_2_HPO_4_, 0.2 g/l MgSO_4_⋅7H_2_O, 0.1 g/l CaCl_2_, and 0.02 g/l FeCl_3_⋅6H_2_O, pH 7.3) with 2 g/l glucose (Wang et al., 2017), or PYS medium (containing the components of both PY6 and Stanier medium) were used to culture *C. hutchinsonii* ATCC 33406 at 30°C. *Escherichia coli* was cultured in Luria-Bertani medium at 37°C. Antibiotic concentrations were as follows: ampicillin (100 μg/ml), chloramphenicol (15 μg/ml), erythromycin (30 μg/ml), and cefoxitin (15 μg/ml).

**TABLE 1:** Strains and plasmids used in this study.

Strains and plasmids	Description^a^	References or source
**Strains**		
*C. hutchinsonii* strains		
ATCC 33406	Wild type	ATCC33406
Δ3238	Deletion mutant of *chu_3238*	This study
C3238	Complementation of Δ3238 mutant	This study
3238-FLAG	Fusion of FLAG-tag at the C-terminal of *chu_3238*	This study
*E. coli* strains		
DH5α	Strain used for plasmid replication	TaKaRa
**Plasmids**		
pSJHC	Gene-targeting template plasmid carrying cat under the control of the *ompA* promoter from *F. johnsoniae*; Ap^*r*^ (Cm^*r*^)	Wang et al., 2014
pSJHS	Gene-targeting template plasmid carrying ermF; Ap^*r*^ (Em^*r*^)	Wang et al., 2014
pCFXSK3328	Plasmid used for deletion of *chu_3328*; Ap^*r*^ (Cf^*r*^)	Guan et al., 2018
pCFXSK3328-3238	Plasmid constructed from pCFXSK3328 used for complementation of Δ3238; Ap^*r*^ (Cf^*r*^)	This study
pSJHS-3238FLAG	Plasmid constructed from pSJHS used for complementation of *chu_3238* with FLAG-tag; Ap^*r*^ (Em^*r*^)	This study

*^a^Antibiotic resistance phenotypes in parentheses are expressed in C. hutchinsonii, and phenotypes not in parentheses are expressed in E. coli (Ap^r^, ampicillin resistance; Cm^r^, chloramphenicol resistance; Em^r^, erythromycin resistance; Cf^r^, cefoxitin resistance).*

**TABLE 2:** Primers used in this study.

Primers	Sequence^a^
3238H1F	TCGCATGCCGTGGTTCCGCTGGTTCG
3238H1R	CGGAGCTCCAAGACGGAAGTTTGTAGG
3238H2F	CGGGTACCCACTTCGATTTACGTTACATTTC
3238H2R	CCGGATCCATGCGTAATAAGGGACAG
3238HF	TCTGCCGATACTAACAAC
3238HR	GTTTGCATAACGACGGTT
T-CM-R	CAGGGATTGGCTGAGACG
3328H1F	TCAAGCATGCAAATATGGTCTGCCCGCGATG
3328H2R	TAATGGATCCGTCCTGAGAAGCCACCAGTGC
3328UF	TGCACAGCTGAATGTACTAC
3328UR	CCTTCTTTGATTATGCCGTC
C3238F	CCGAGCTCCATTTACCACCAATACC
C3238R	TGGTCGACCAAATGTATGCAGCTTGT
T-EM-R	TGAACAGTAAGAAACCCCTTGCCT
3238CH1F	CGTCTAGACTATTGTTACTACAACAACCATCAATTCC
3238-FLAG-R1	CATCCTTGTAATCATTATCTATAACAGAATCATCAATTTG
3238-FLAG-R2	GCGAGCTCTTACTTATCGTCGTCATCCTTGTAATCATTAT
3238CH2F	GGGGTACCTGACACACATACTAGACAGGACG
3238CH2R	CGGGATCCACATGTAGATGTAATCAAAAATGTG
3238YZ-F	GCATGTTGCCAACCTCAC
3238YZ-R	AAGCAACACCCGCCACAT
3238-FLAG-F	CCAAGCAAGTGGGATAGT
3238-YZ	ATGGCTTATCTGATACCG

*^a^Restriction sites in sequence are underlined.*

### Gene Targeting of *chu_3238*

The double-crossover homologous recombination method, using the plasmid pSJHC as described by Wang et al. (2014), was used to delete the *chu_3238* gene. The ∼2.8-kb 3238H1 fragment, spanning two flanking genes (the last 1,843 bp of *chu_3237* and the first 715 bp of *chu_3238*) was amplified using the primers 3238H1F and 3238H1R. The 3238H1 fragment was inserted into pSJHC plasmid digested with *Sph*I and *Sac*I, to generate pSJHC-H1. The ∼2.2-kb 3238H2 fragment, spanning five flanking genes (the downstream 70 bp of *chu_3238*, *chu_3239*, *chu_3240*, and *chu_3241* and the last 29 bp of *chu_3242*) was amplified using primers 3238H2F and 3238H2R. 3238H2 fragment was ligated into pSJHC-H1 digested with *Kpn*I and *Bam*HI to produce pSJHC-H1H2. Using pSJHC-H1H2 as the template, primers 3238H1F and 3238H2R were used to amplify a fragment that contained 3238H1, 3238H2, and the chloramphenicol resistance gene. This was transformed into competent cells of *C. hutchinsonii* as previously described (Xu et al., 2012). The transformants cultured at 30°C on PYS agar medium (10 g/l agar) containing chloramphenicol were verified using primers 3238HF/3238HR and 3238HF/T-CM-R. The *chu_3238* deletion mutant was named Δ3238.

### Complementation of *chu_3238* in the Δ3238 Mutant

The plasmid pCFXSK3328 was used to complement the *chu_3238* gene as described by Guan et al. (2018). Briefly, the ∼1.6-kb complemented fragment, C-3238, containing *chu_3328* and its native promoter (from the upstream 324 bp and the downstream 114 bp of *chu_3238*) was amplified using primers C3238F and C3238R. C-3238 was inserted into pCFXSK3328 between the *Sal*I and *Sac*I sites, generating pCFXSK3328-3238. The PCR product that contained the homologous arms of *chu_3328*, C-3238, and the cefoxitin resistance gene was amplified with primers 3328H1F and 3328H2R and transformed into competent cells of Δ3238. The transformants were grown at 30°C on PYS agar medium supplemented with chloramphenicol and cefoxitin. C-3238 fragment was integrated into the pseudogene of *chu_3328* in the Δ3238 mutant genome, which was verified by PCR using the primers 3328UF/3328UR and 3328UF/C3238R. The complemented strain was named C3238.

### Determination of the Growth Ability

Growth curves were generated by harvesting an equal amount of mid-exponential phase cells of the wild type (WT), the Δ3238 mutant, and C3238 cultured in PYS and using these cells to inoculate PY6 medium, Stanier medium, and PYS medium. The PY6 medium was supplemented with 1 g/l KNO_3_, 1 g/l K_2_HPO_4_, 0.2 g/l MgSO_4_⋅7H_2_O, 0.1 g/l CaCl_2_, or 0.02 g/l FeCl_3_⋅6H_2_O, respectively, to determine the effect of ions on growth. In addition, all media was supplemented with 4 g/l glucose as the carbon source. Absorbance at 600 nm was monitored using a Bioscreen C analyzer (Oy Growth Curves Ab Ltd., Helsinki, Finland). Cellulose degradation ability was assessed by spotting the cells on a filter paper on Stanier agar and then incubating the plates at 30°C to observe filter paper degradation.

### Determination of Colony Spreading

Soft agar (2 g/l peptone, 0.5 g/l yeast extract, 2 g/l glucose, and 5 g/l agar) and hard agar (2 g/l peptone, 0.5 g/l yeast extract, 1 g/l glucose, and 10 g/l agar) were used to determine colony spreading. An equal amount of mid-exponential phase cells was spotted on soft agar or on hard agar and incubated at 30°C. Colony spreading was observed as described by Guan et al. (2018).

### Determination of Cellulase Activity

Cellulase activity was determined as previously described (Zhao et al., 2020). For intact cell samples, mid-exponential phase cells were collected at 5,000 × *g* for 10 min and suspended in Na_2_HPO_4_-KH_2_PO_4_ buffer (100 mM, pH 6.8). To lyse the cells, 2% (vol/vol) Triton X-100 was added to the cell samples in Na_2_HPO_4_-KH_2_PO_4_ buffer at 4°C for 2 h. This was then used as the cell extract sample. Endoglucanase activity was determined using sodium carboxymethyl cellulose (CMC-Na) as the substrate. An equal volume (500 μl) of the sample and 1% (wt/vol) CMC-Na were mixed and incubated for 30 min at 30°C; 200 μl of dinitrosalicylic acid (DNS) was added, and the mixture was boiled for 10 min to stop the enzyme activity. The mixture was cooled on ice and centrifuged at 12,000 × *g* for 5 min. The concentration of the reducing ends in the supernatant was determined by measuring the absorbance at 550 nm (Miller, 1959). The protein concentration, used as an indicator of biomass, was determined as described by Bradford (1976).

### Protein Fractionation of the Bacterial Cultures

Extracellular and outer membrane fractions were prepared as previously described (Wang et al., 2014; Zhao et al., 2020). The WT and the Δ3238 mutant were cultured in PY6 medium. The subsequent steps were performed at 4°C. The mid-exponential phase cells were collected by centrifugation at 5,000 × *g* for 10 min, and the residual cells in the supernatants were removed using a 0.22-μm polyvinylidene difluoride (PVDF) filter. The supernatants were pelleted with 10% (wt/vol) trichloroacetic acid (TCA) for 2 h. Extracellular proteins were obtained by centrifugation at 12,000 × *g* for 10 min. For the outer membrane proteins, pre-cooled 50 mM piperazine-N,N′-bis (2-ethanesulfonic acid) (PIPES) buffer (pH 6.8) containing 0.5 M NaCl was used to resuspend the cell pellets that were collected in the first step. The suspension was stirred at 150 rpm for 15 min and then centrifuged at 12,000 × *g* for 20 min. The supernatant was ultracentrifuged at 100,000 × *g* for 20 min. The outer membrane protein pellets were resuspended in pre-cooled PIPES buffer. Periplasmic proteins were prepared as previously described by Soares et al. (2003) with some modifications. The mid-exponential phase cell pellets were resuspended in pre-cooled 10 mM Tris-HCl (1 mM EDTA, pH 7.5) containing 20% (wt/vol) sucrose and incubated for 10 min. Then, the cells were centrifuged at 7,000 × *g* for 10 min, and the pellet was resuspended with pre-cooled ultra-pure water, with incubation for 10 min. The suspension was centrifuged at 7,000 × *g* for 10 min, and the periplasmic proteins in the supernatant were further concentrated with an ultrafiltration centrifugal tube (Millipore, MA, United States). Equal amounts of proteins as determined by the Bradford method described above were separated by sodium dodecyl sulfate–polyacrylamide gel electrophoresis (SDS-PAGE) and stained using Coomassie brilliant blue R-250. Protein differences between the WT and the Δ3238 mutant were identified by liquid chromatography-tandem mass spectrometry (LC-MS/MS).

### Determination of Cellulose-Binding Ability

In order to observe cells on cellulose fiber, mid-exponential phase cells of the WT and the Δ3238 mutant were inoculated onto a filter paper on Stanier agar medium and incubated for 48 h. The filter paper with the cells was then fixed with 2.5% (vol/vol) glutaraldehyde at 4°C overnight. The cells fixed onto the filter paper were washed twice by soaking in phosphate-buffered saline (PBS, pH 7.2) and dehydrated with ethanol. The filter paper was dried and observed using scanning electron microscopy (SEM) (Ji et al., 2013).

The cellulose-binding rate of cells was determined by collecting the mid-exponential phase cells by centrifugation at 5,000 × *g* for 10 min and washing with phosphate buffer (137 mM NaCl, 2.7 mM KCl, 10 mM Na_2_HPO_4_, and 2 mM KH_2_PO_4_, pH 7.4). The cells were then resuspended with phosphate buffer to adjust the suspension with the absorbance at 600 nm to 1.0. To the 3.5 ml of cell suspension, 0.5 ml of 10% (wt/vol) sterilized cellulose (Aladdin, China) was added, and the mixture was shaken at 100 rpm at 30°C for 10 min. Cellulose with binding cells settled when the mixture was stand for 1 h. The cells that did not bind to cellulose was in the supernatant, which was measured by the absorbance at 600 nm. Cellulose-binding rate was calculated as described by Ji et al. (2012). Percentage of adhesion was measured using the equation: (1 − OD_600_) × 100, where OD_600_ is the absorbance of cells that did not bind cellulose at 600 nm.

### Examination and Extraction of Lipopolysaccharides

To examine the production of extracellular polysaccharides, a 10-fold dilution of samples of mid-exponential phase cells were inoculated on Stanier agar containing 30 μg/ml Congo red and cultured at 30°C for 3 days in the dark.

Lipopolysaccharides (LPS) were isolated by hot aqueous-phenol as described by Davis and Goldberg (2012), with some modifications. Briefly, 2 × SDS buffer (0.1 M Tris-HCl, pH 6.8) with 4% (vol/vol) β-mercaptoethanol, 4% (wt/vol) SDS, 20% (vol/vol) glycerol, and bromophenol blue was prepared. SDS buffer (1×) was prepared by diluting the 2 × SDS buffer with sterile distilled water. Mid-exponential phase cells were suspended in 200 μl of 1 × SDS buffer. The cells were then boiled for 15 min and cooled to room temperature. Five microliters each of DNaseI solution (10 mg/ml) and RNase solution (10 mg/ml) were added to the samples, followed by incubation at 59°C for 3 h for nucleic acid and protein digestion. Precooled Tris-saturated phenol (200 μl) was added, and the samples were incubated at 65°C for 15 min. Each sample was vortexed occasionally. After cooling the samples to room temperature, 1 ml of diethyl ether was added with vigorous shaking. The samples were centrifuged at 20,600 × *g* for 10 min. The bottom blue layer was carefully removed from the extracted LPS, and 2 × SDS for separating LPS by Tris-Tricine-SDS-PAGE was added. The bands of LPS were observed by silver staining using a Fast Silver Stain Kit (Beyotime, China).

### Disk Diffusion Susceptibility Assay

The disk diffusion assay was performed as described by Liu et al. (2016). The mid-exponential phase cells were mixed with Stanier medium containing 5 g/l agar and 2 g/l glucose, and the mixture was poured onto a layer of Stanier medium containing 10 g/l agar and 2 g/l glucose. A sterilized 7-mm paper disk was placed on the agar plate. Five microliters of each of 1% (wt/vol) violet, 2 M dithiothreitol (DTT), 20 μg/ml ampicillin (Ap), and 0.5 M cumene hydroperoxide (CHP) were separately added to the paper disks. The agar plates were incubated at 30°C for 3 days. The growth inhibition zones surrounding the paper disk were recorded.

### Subcellular Location Assay

To localize CHU_3238, we fused a FLAG epitope tag to the C-terminus of CHU_3238, as previously described (Zhao et al., 2020). Briefly, the WT genome was used as a template. A 2.5-kbp fragment named 3238CH2 was amplified with primers 3238CH2F and 3238CH2R. To generate pSJHS-3238CH2, 3238CH2 was inserted between the *Bam*HI and *Kpn*I sites of plasmid pSJHS. The FLAG sequence was fused to the C-terminus of *chu_3238* using primers 3238CH1F/3238-FLAG-R1 and 3238CH1F/3238-FLAG-R2 by two-step PCR to obtain the fragment 3238CH1-FLAG. To produce pSJHS-3238FLAG, 3238CH1-FLAG was ligated into the *Xba*I and *Kpn*I sites of pSJHS-3238CH2. Using pSJHS-3238FLAG as the template, the PCR product 3238CH1H2-FLAG was amplified with primers 3238CH1F and 3238CH2R and transformed into the competent cells of the WT strain. The transformants were cultured on PY6 medium containing 10 g/l agar and erythromycin at 30°C and were verified using primers 3238YZ-F/3238YZ-R. An appropriate transformant was identified and named 3238-FLAG.

Mid-exponential phase cells of 3238-FLAG and the WT, cultured in PY6 medium, were collected to prepare the outer membrane proteins and total membrane proteins, as previously described (Yang et al., 2016). Equal amounts of the outer membrane and total membrane proteins were separated by SDS-PAGE. CHU_3238 was identified by western blotting using an anti-FLAG mouse monoclonal antibody (Wang et al., 2014).

### Co-immunoprecipitation With 3238-FLAG

The 3238-FLAG strain was used to pull down the interacting proteins by co-immunoprecipitation. Total membrane proteins of 3238-FLAG and the WT were prepared as described above and were incubated with 2% (wt/vol) n-dodecyl β-D-maltoside at 4°C for 2 h. The samples were then centrifuged at 12,000 × *g* for 10 min at 4°C. The supernatant contained the total dissolved membrane proteins. Co-immunoprecipitation (Co-IP) was carried out using an anti-FLAG affinity gel (Dai-An Biotech, China) according to the manufacturer’s instructions. The proteins bound to the gel were boiled with 5 × SDS-PAGE loading buffer for 10 min and then centrifuged at 12,000 × *g* for 5 min. The eluted proteins in the supernatant were separated by SDS-PAGE and visualized by staining the gel with Coomassie brilliant blue R-250. The proteins in 3238-FLAG that were different from the WT were sliced and identified by LC-MS/MS.

## Results

### CHU_3238 Was Predicted to Be PorV by Bioinformatics Analysis

**FIGURE 1:**
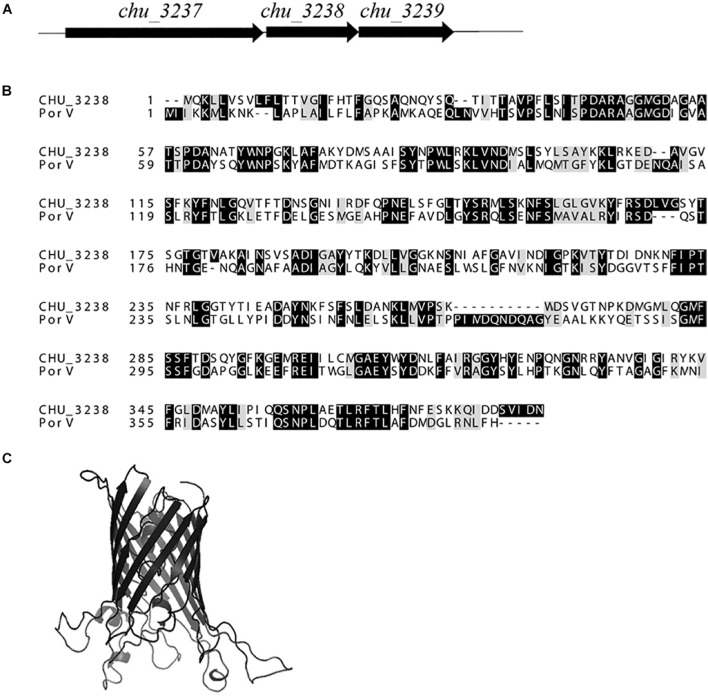
Alignment of CHU_3238 with PorV. **(A)** Illustration of *chu_3238* and the surrounding genes. **(B)** Alignment of CHU_3238 and PorV from *P. gingivalis* using MUSCLE. Similar amino acids are marked by light shading. **(C)** Predicted SWISS-MODEL three-dimensional structure of CHU_3238.

As shown in Figure 1, the gene upstream of *chu_3238* is *chu_3237*. *chu_3237* is reported to function as a *porU* in the T9SS (Wang et al., 2014). In *P. gingivalis*, the downstream portion of *porU* is *porV*. The protein CHU_3238 from *C. hutchinsonii* showed 38.6% sequence identity with PorV. Downstream of *chu_3238* is *chu_3239*, which encodes CHU_3239, annotated as a zinc protease. A structural model of CHU_3238 was constructed following the three-dimensional molecular structure of PorV, an outer membrane β-barrel shuttle protein from *F. johnsoniae*. The N-terminal signal peptide of CHU_3238 is at residues 1–21 predicted by Phobius, and the location of CHU_3238 is predicted in the outer membrane by PSORTb. A bioinformatics analysis indicated that CHU_3238 may be a PorV, which is essential for CTD protein secretion and localization in the T9SS (Kharade and McBride, 2015; Glew et al., 2017).

### The Δ3238 Mutant Showed Significant Phenotypic Differences From the Wild Type

**FIGURE 2:**
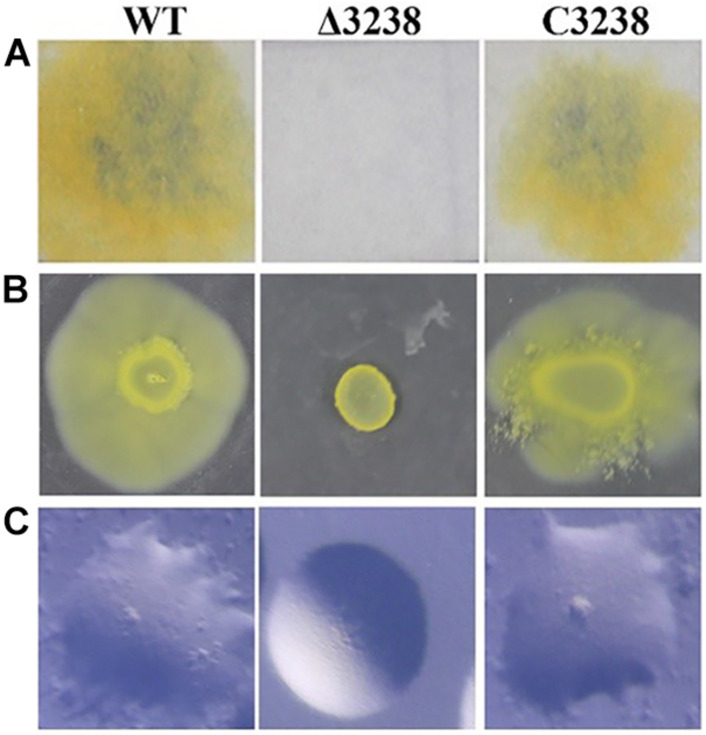
Cellulose degradation and colony spreading of the wild type (WT), the Δ3238 mutant, and C3238 cells. **(A)** Filter paper degradation on Stanier agar. **(B)** Colony spreading on soft agar. **(C)** Colony spreading on PY2 hard agar. All experiments were performed in triplicate.

To study the role of *chu_3238* in *C. hutchinsonii*, it was disrupted by homologous recombination (Supplementary Figure 1A). The Δ3238 mutant was successfully constructed by verification with PCR (Supplementary Figure 1B). The complemented strain C3238 was constructed by expressing *chu_3238* with its native promoter in the pseudogene *chu_3328* in the Δ3238 mutant, which was verified by PCR (Supplementary Figure 1C). As shown in Figure 2, the Δ3238 mutant completely lost the ability to degrade cellulose and to spread on soft and hard agar. In contrast, the complemented strain C3238 could degrade cellulose and spread on soft and hard agar in a similar way to the WT.

**FIGURE 3:**
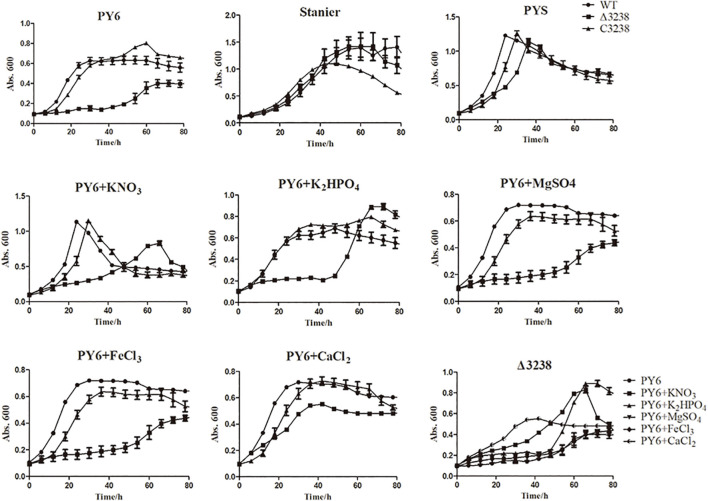
Growth curves of the wild type (WT), the Δ3238 mutant, and the complemented strain, C3238. The cells were cultured in PY6; Stanier; PYS; and PY6 with KNO_3_, K_2_HPO_4_, MgSO_4_, FeCl_3_, or CaCl_2_, with 0.4% (*w*/*v*) glucose added as the sole carbon source. Cells were grown at 30°C. The measurement was performed in triplicate, and error bars indicate standard error.

Transformants of the *chu_3238* deletion mutant were cultured in PYS agar medium that combined both the PY6 and Stanier medium, as no transformants were grown in PY6 agar medium for 2 weeks. As shown in Figure 3, the Δ3238 mutant showed an obvious and long lag phase in PY6 medium compared with the WT, while it had the same growth ability as the WT in the Stanier medium. When the PY6 medium and Stanier medium were mixed as PYS medium, the growth of the Δ3238 mutant could be restored even though the Δ3238 mutant showed a slightly longer lag phase than the WT. C3238 could complement the growth defect of the Δ3238 mutant in PY6 medium. The growth curves in PY6 medium supplemented with KNO_3_, K_2_HPO_4_, MgSO_4_, FeCl_3_, or CaCl_2_ were plotted. The results showed that CaCl_2_ could efficiently shorten the lag phase of the Δ3238 mutant in PY6 medium, but did not reach the same biomass as the WT. The addition of KNO_3_ or K_2_HPO_4_ to the PY6 medium improved the resultant biomass of the Δ3238 mutant; however, the lag phase could not be shortened.

### Deletion of *chu_3238* Decreased Endoglucanase Activity

**FIGURE 4:**
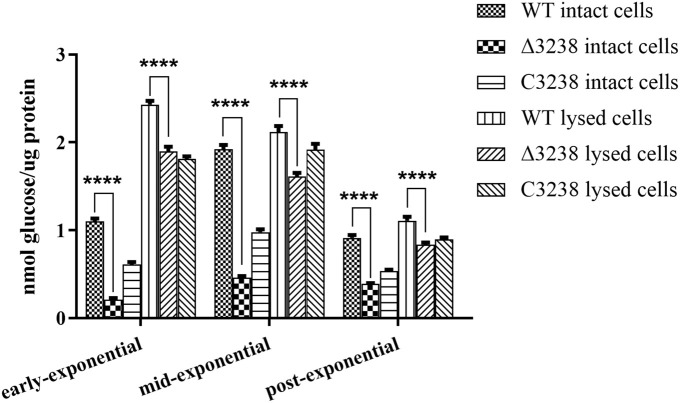
Endoglucanase activities of the wild type (WT), the Δ3238 mutant, and the complemented strain C3238. Endoglucanase activity was determined using sodium carboxymethyl cellulose (CMC-Na) as the substrate, and the reducing sugars were measured using the 3,5-dinitrosalicylic acid procedure. The measurement was performed in triplicate, and error bars indicate standard error. Significance reported as ****p < 0.0001.

*Cytophaga hutchinsonii* has 18 predicted endoglucanases (Taillefer et al., 2018), of which 12 are predicted CTDs, which are thought to be extracellular or outer membrane proteins. We deduced that the T9SS might aid in the secretion of endoglucanases with CTDs. Therefore, the endoglucanase activities of the WT and the Δ3238 mutant were determined. As shown in Figure 4, the endoglucanase activity of intact cells of the Δ3238 mutant was significantly decreased and displayed a 70% reduction compared to the WT. The endoglucanase activity of lysed cells of the Δ3238 mutant exhibited a 20% reduction compared to that of the WT (average from three growing phases). The decreased endoglucanase activity of intact cells suggests that secretion of the cell surface endoglucanases was affected in the Δ3238 mutant.

### Deletion of *chu_3238* Resulted in Abnormal Protein Secretion

**FIGURE 5:**
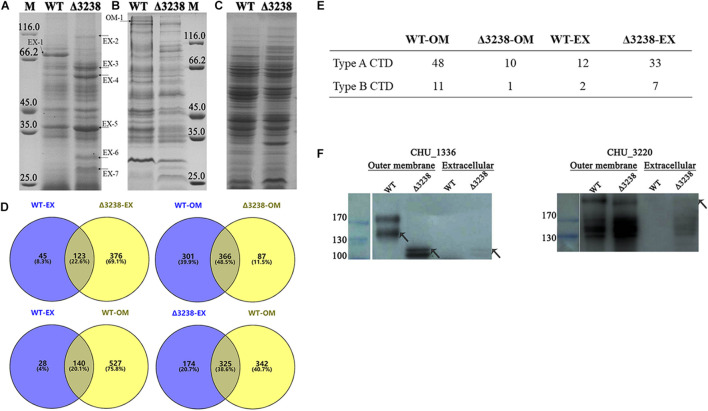
Differences between the wild type (WT) and the Δ3238 mutant in extracellular **(A)**, outer membrane **(B)**, and periplasmic proteins **(C)**. The different proteins are marked with arrowheads. M, protein marker (kDa). **(D)** Comparison of extracellular (EX) and the outer membrane (OM) proteins between the wild type (WT) and the Δ3238 mutant by proteomic analysis. **(E)** The number of type A and type B CTD proteins in the extracellular space and the outer membrane of the WT and the Δ3238 mutant. **(F)** Western blot was performed with anti-CHU_1336 serum and anti-CHU_3220 serum to detect CHU_1336 (Left) and CHU_3220 (Right) in the extracellular space and the outer membrane of the WT and the Δ3238 mutant. All experiments were performed in triplicate.

To identify the protein secretion ability of the Δ3238 mutant, extracellular proteins, outer membrane proteins, and periplasmic proteins of the WT and the Δ3238 mutant were prepared. As shown in Figure 5A, the extracellular proteins of the Δ3238 mutant showed a higher intensity compared to the WT, except for the CTD protein CHU_0344 (band EX-1), which was absent in the Δ3238 mutant. The visibly increased extracellular proteins in the Δ3238 mutant (marked with arrowheads in the figure) were excised and identified by LC-MS/MS. Most of them did not have CTD and did not belong to the extracellular proteins as predicted by PSORTb (Table 3). The outer membrane proteins of the Δ3238 mutant decreased (Figure 5B). As shown in Figure 5D, the proteomic analysis by LC-MS/MS showed that 168 proteins were identified in the extracellular medium of the WT (Supplementary Data Sheet 1), and 499 proteins were identified in the extracellular medium of the Δ3238 mutant (Supplementary Data Sheet 2). Among them, 123 proteins were identified in both the WT and the Δ3238 mutant, suggesting that 376 proteins may be abnormally secreted into the extracellular medium of the Δ3238 mutant. A total of 667 proteins were identified in the outer membrane of the WT strain (Supplementary Data Sheet 3), and 453 proteins were identified in the outer membrane of the Δ3238 mutant (Supplementary Data Sheet 4). Among them, 366 proteins were common in the WT and the Δ3238 mutant, suggesting that 87 proteins were abnormally secreted, and 301 proteins were absent in the outer membrane of the Δ3238 mutant. The extracellular and outer membrane of the WT contained a total of 140 proteins that were common between the two. A total of 325 proteins overlapped between the extracellular region of the Δ3238 mutant and the outer membrane of the WT. Moreover, the WT was identified to express 12 type A CTD proteins and two type B CTD proteins in the extracellular space and 48 type A CTD proteins and 11 type B CTD proteins in the outer membrane, while the Δ3238 expressed 33 type A CTD proteins and seven type B CTD proteins in the extracellular space and 10 type A CTD proteins and one type B CTD proteins in the outer membrane (Figure 5E), suggesting the secretion of type A and type B CTD proteins was affected in the Δ3238 mutant. CHU_1075, a type A CTD protein, identified in the outer membrane of the WT (band OM-1), was identified in the extracellular region of the Δ3238 mutant. Western blot was done with anti-CHU_1336 (type A protein) serum and anti-CHU_3220 (type B protein) serum (Figure 5F). The result showed that CHU_1336 in the WT outer membrane with an increased molecular weight was not detected in the Δ3238 outer membrane. The theoretical molecular weight of CHU_1336 was 105 kDa, which was detected in the outer membrane and extracellular space in the Δ3238 mutant. CHU_3220 was detected both in the WT and Δ3238 outer membrane, and it was also detected in the extracellular space of the Δ3238 mutant. In *P. gingivalis*, a weakened protein secretion ability caused abundant CTD proteins in the periplasm in the PorV mutant (Chen et al., 2011). However, the periplasmic proteins of the Δ3238 mutant may be not excessively accumulated compared to the WT (Figure 5C). These results suggest that the outer membrane proteins were abnormally secreted into the extracellular medium in the Δ3238 mutant.

**TABLE 3:** Identification of the marked extracellular and outer membrane proteins by LC-MS/MS^α^.

Band	CHU no.	Mw (kDa)	Protein description and/or name	CTD	Location
OM-1	CHU_1075	271.4	Glycoside hydrolase family 8 protein	Yes	EX
EX-1	CHU_0344	95.2	Hypothetical protein	Yes	EX/OM
EX-2	CHU_1230	110.7	Zinc protease	No	Unknown
EX-3	CHU_0134	55.2	Thiol-disulfide isomerase	No	Unknown
EX-3	CHU_0268	66.8	Hypothetical protein	Yes	OM
EX-4	CHU_1608	58.6	Hypothetical protein	Yes	EX/OM
EX-5	CHU_1993	29.6	Hypothetical protein	No	Unknown
EX-6	CHU_2724	23.1	Alkyl hydroperoxide reductase	No	CP
EX-7	CHU_2699	24.8	Gliding motility-related protein	No	OM

*^α^ Mw is the theoretical molecular mass. CTD is the conserved domain in the protein that were identified and transported by the T9SS. The location is predicted with pSORTb 3.0. EX, extracellular; OM, outer membrane; CP, cytoplasmic.*

### The Weakened Cellulose-Binding Ability of the Δ3238 Mutant

**FIGURE 6:**
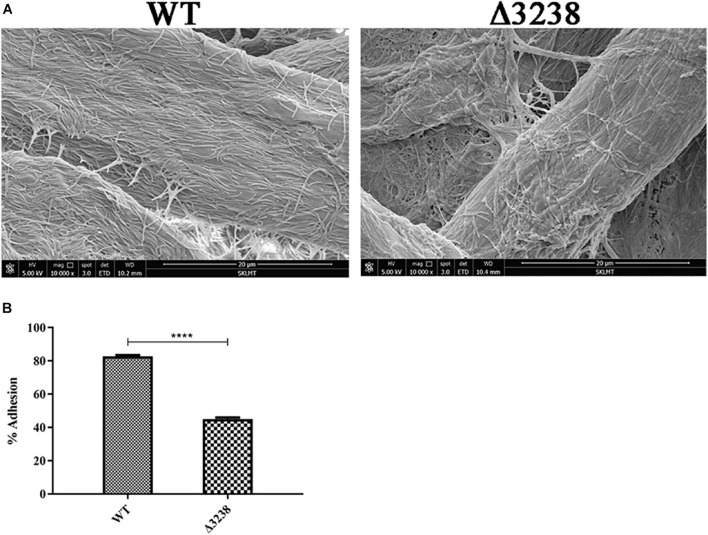
Cellulose-binding ability of the wild type (WT) and the Δ3238 mutant. **(A)** Scanning electron microscopy (SEM) images showing the arrangement of the WT and the Δ3238 mutant on cellulose fibers. **(B)** Adhesion of the WT and the Δ3238 mutant to Avicel. The experiments were performed in triplicate, and the error bars indicate standard error. Significance reported as ****p < 0.0001.

Cellulose-binding ability is mainly dependent on outer membrane proteins in *C. hutchinsonii.* Since the outer membrane proteins in the Δ3238 mutant were decreased, this may be a cause for the reduced ability of the cells to bind cellulose. As shown in Figure 6A, SEM revealed that the WT cells were arranged regularly on the cellulose fibers, whereas there were only a few of the Δ3238 mutant cells on the cellulose fibers. The cellulose-binding rate of the Δ3238 mutant was decreased by approximately 30% compared to that of the WT (Figure 6B), suggesting that a decreased presence of the outer membrane proteins affected the cellulose-binding ability in the Δ3238 mutant.

### Change of Cell Integrity in the Δ3238 Mutant

**FIGURE 7:**
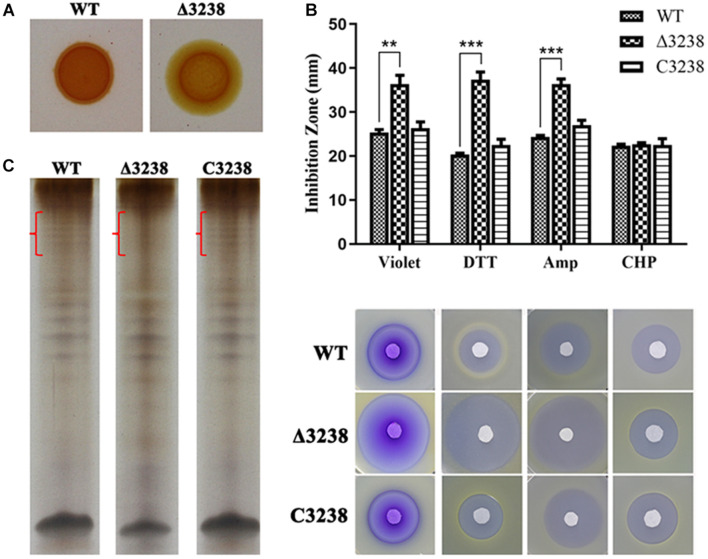
Biological consequences of the deletion of *chu_3238*. **(A)** The wild type (WT), the Δ3238 mutant, and the C3238 cells inoculated on Stanier agar with 30 μg/ml Congo red. **(B)** Extraction of lipopolysaccharides (LPS) by the hot aqueous-phenol method and visualization with silver stain. Difference of LPS among WT, the Δ3238 mutant, and C3238 was marked with red brace. **(C)** Disk diffusion susceptibility assay for the WT and the Δ3238 mutant. DTT, dithiothreitol; Amp, ampicillin; CHP, cumene hydroperoxide. All experiments were performed in triplicate, and the error bars indicated standard errors. Significance reported as **p < 0.01, ***p < 0.001.

Previous studies have reported that PorV is essential for the O-deacylation of LPS. We examined the cell-surface LPS with Congo red and extracted them from the WT and the Δ3238 mutant. The cell surface of the Δ3238 mutant was visibly pale when stained with Congo red (Figure 7A). LPS was separated by Tris-Tricine-SDS-PAGE, indicating that the *O*-antigen of LPS in the Δ3238 mutant was partially lost. The integrity of the LPS was disrupted due to deletion of *chu_3238* (Figure 7B).

We determined cell sensitivity to toxic reagents, including crystal violet, ampicillin, DTT, and CHP. As shown in Figure 7C, the Δ3238 mutant cells were more sensitive to these reagents except CHP. CHP is a kind of oxidant, and DTT is a kind of reductant. There was no obvious difference in CHP between WT and the Δ3238 mutant, and the Δ3238 mutant was more sensitive to DTT, suggesting that the Δ3238 mutant could resist the oxidant but not resist the reductant. The complemented strain C3238 restored the LPS production, as well as the resistance to these reagents, to the same level observed in the WT strain, suggesting that the deletion of *chu_3238* increased outer membrane permeability.

### Subcellular Location of CHU_3238 and Its Co-immunoprecipitated Proteins

**FIGURE 8:**
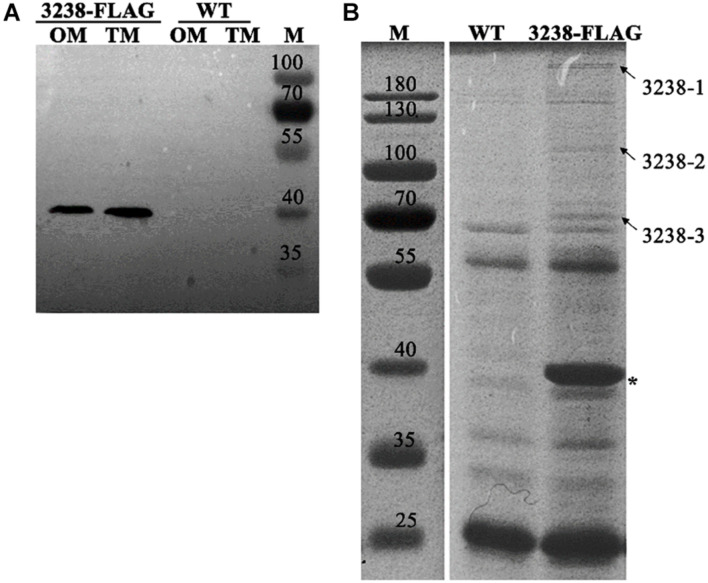
Subcellular location of CHU_3238 and co-immunoprecipitation with 3238-FLAG. **(A)** Western blot of outer membrane (OM) and total membrane (TM) proteins of 3238-FLAG. **(B)** Co-immunoprecipitation with 3238-FLAG using anti-Flag affinity gel. The arrowheads indicate the different proteins between the WT and 3238-FLAG identified by LC-MS/MS. The asterisk indicates CHU_3238 with the FLAG tag. M, protein marker, kDa. The wild type (WT) was used as the negative control. All experiments were performed in triplicate. The full-size SDS-PAGE gel and PVDF membrane are shown in Supplementary Figures 4, 5.

CHU_3238 was predicted to be an outer membrane protein as it was homologous to the outer membrane protein, PorV. We constructed the 3238-FLAG strain, in which the C-terminus of CHU_3238 was inserted into a FLAG-tag (Supplementary Figure 2A). The 3238-FLAG strain was confirmed by PCR (Supplementary Figure 2B). CHU_3238 was detected in both the outer membrane and the total membrane fractions (Figure 8A). Considering the predicted sequence characteristics and structural model of CHU_3238, detected in the outer membrane of the WT by proteomic analysis (Supplementary Data Sheet 3), we deduced that CHU_3238 was localized to the outer membrane of in *C. hutchinsonii*.

PorV has multiple roles in the T9SS. It functions as a shuttle outer membrane protein that interacts with the T9SS components to transport cargo proteins from the periplasm to the outer membrane, and it can anchor the C-terminal signal peptidase (PorU) through non-covalent interactions (Glew et al., 2017; Lauber et al., 2018). We used the 3238-FLAG strain to prepare cell extracts in which the proteins interacting with CHU_3238 were purified using an anti-FLAG affinity gel (marked with an asterisk in Figure 8B). Specific co-purified proteins (marked with arrowheads) were identified by LC-MS/MS and are listed in Table 4. Homologs of a T9SS component, SprA (CHU_0029), and a TonB-dependent receptor (CHU_1606) were identified. The translocon SprA forms a complex with PorV and the Plug protein to form the transport channel in *F. johnsoniae* (Lauber et al., 2018). The TonB-dependent receptor is responsible for Fe transport and has a Plug domain that acts as the channel gate. In addition, CHU_1606 showed 32.8% sequence identity in amino acid with PG0534 (PorF, a TonB-dependent receptor), a T9SS component in *P. gingivalis*. PG0534 was required for normal secretion of gingipains in *P. gingivalis* (Saiki and Konishi, 2010; Lasica et al., 2017; Gorasia et al., 2020a). Therefore, *C. hutchinsonii* may form the complex to transport the T9SS cargo proteins.

**TABLE 4:** Identification of the co-immunoprecipitated proteins with CHU_3238 by LC-MS/MS^α^.

Band	CHU no.	Mw (kDa)	Protein description and/or name	Location
3238-1	CHU_0029	268.4	SprA	CM/OM
3238-2	CHU_1606	91.1	TonB-dependent receptor	OM
3238-3	CHU_0029	268.4	SprA	CM/OM

*^α^ Mw is the theoretical molecular mass. The location is predicted with pSORTb 3.0. CM, cytoplasmic membrane. OM, outer membrane.*

## Discussion

*Cytophaga hutchinsonii* efficiently degraded cellulose and rapidly glided over the surface. However, both mechanisms are still under investigation. The T9SS is widely spread and confined to the phylum *Bacteroidetes*, and many gliding-related proteins in *F. johnsoniae* belong to the components of the T9SS. The T9SS is involved in protein secretion and gliding motility and has been implicated in some bacterial diseases in many members of *Bacteroidetes*. It secretes cargo proteins with specific CTDs to the cell surface and anchors them with glycolipids (Slakeski et al., 2011; Gorasia et al., 2015; Veith et al., 2020). *C. hutchinsonii* contains all the homologous proteins of the T9SS and most of the gliding-related genes. Although some of the components of the T9SS have been found to affect cellulose degradation and the gliding ability in *C*. *hutchinsonii*, the mechanism as to how the cargo proteins are transported and the functional differences of the T9SS compared to other members of the *Bacteroidetes* phylum need to be further explored. In this study, we deleted a protein, CHU_3238, which exhibited 38.6% amino acid sequence identity with PorV, a shuttle outer membrane protein involved in protein secretion and modification in the T9SS (Chen et al., 2011; Glew et al., 2017).

The PorV deletion mutant in *F. johnsoniae* does not affect gliding motility because the gliding motility adhesion protein, SprB, does not require PorV for secretion (Kharade and McBride, 2015). The PorV mutant in *Flavobacterium columnare* also retains its ability to glide (Barbier et al., 2020). The Δ3238 mutant showed definite gliding defects on soft and hard agar. *C. hutchinsonii* expresses the SprB homolog (CHU_2225), but it is not necessary for gliding motility (data not shown). It is possible that the gliding mechanisms in the phylum *Bacteroidetes* are different, and *C. hutchinsonii* secretes other adhesions through the T9SS that affects gliding motility. In addition, the Δ3238 mutant showed growth defect in the PY6 medium, while it showed almost the same growth ability as the WT in the Stanier medium. Further investigation of the cell growth showed that CaCl_2_ had the ability to shorten the lag phase in the PY6 medium but could not reach the same biomass as the WT. KNO_3_ and K_2_HPO_4_ could improve the biomass of the Δ3238 mutant in the PY6 medium but could not shorten the lag phase. These results suggest that Ca^2+^ and K^+^ might be important for Δ3238 mutant growth. Studies from our group have shown that *chu_0174* (*gldN*), *chu_0029* (*sprA*), and *chu_2709* (*sprT*) deletion mutants in *C. hutchinsonii* had growth defects in Ca^2+^- and Mg^2+^-deficient media (Gao et al., 2020, 2021). *chu_3238* may also play a role in ion assimilation of Ca^2+^ and K^+^, but not in the assimilation of Mg^2+^. Ion assimilation was not found in the T9SS components of other *Bacteroidetes*, and deletion of the *porU* homolog (*chu_3237*) or the *sprP* homolog (*chu_0170*) in *C. hutchinsonii* did not cause growth defects in PY6 medium. The difference in ion assimilation observed among these T9SS components suggests their functional diversity.

The protein secretion ability of the Δ3238 mutant was abnormal. The extracellular proteins were increased while the outer membrane proteins were decreased in the Δ3238 mutant compared with those of the WT (Figure 5); this was further confirmed by LC-MS/MS. Among the 499 extracellular proteins in the Δ3238 mutant (Supplementary Data Sheet 2), 376 proteins were not detected in the extracellular medium of WT (Supplementary Data Sheet 1), while 325 proteins overlapped with the outer membrane proteins of the WT (Supplementary Data Sheet 3). Moreover, 14 CTD proteins were identified as extracellular fraction and 59 CTD proteins were identified in the outer membrane fraction of the WT strain. In contrast, 40 CTD proteins were identified in the extracellular fraction and 13 CTD proteins in the outer membrane fraction of the Δ3238 mutant, containing more type A CTD proteins and less type B CTD proteins. CHU_1075, a type A CTD protein, identified in the outer membrane of the WT strain (band OM-1), was found in the extracellular region of the Δ3238 mutant. Western blot showed that CHU_1336 (a type A protein) in the WT outer membrane with an increased modified molecular weight was not detected in the Δ3238 outer membrane, while it was detected in the outer membrane and extracellular space in the Δ3238 mutant with the theoretical molecular weight. CHU_3220 was detected both in the WT and Δ3238 outer membrane, and it was also detected in the extracellular space of the Δ3238 mutant. These suggest that the type A proteins and the type B proteins in the Δ3238 mutant were modified with a different mechanism. We suggest that the secreted proteins, including the CTD proteins, could not be anchored to the outer membrane and were released into the extracellular medium. However, a CTD protein identified in the extracellular region of the WT, CHU_0344 (band EX-1), was not found in the extracellular region of the Δ3238 mutant, which could not be explained and requires an investigation. In addition, the Δ3238 mutant could utilize cellobiose as the sole carbon source (Supplementary Figure 3), suggesting that the β-glucosidases were not affected. However, only about 30% endoglucanase activity was retained in the intact cells of the Δ3238 mutant compared to the WT. There are 18 predicted endoglucanases in *C. hutchinsonii*, and 12 endoglucanases have CTDs that were identified by the T9SS. These endoglucanases were identified in the extracellular fraction or in the outer membrane fraction, except for the two identified in the inner membrane fraction (Taillefer et al., 2018). A proteomic analysis showed that 12 endoglucanases were decreased or absent in the outer membrane of the Δ3238 mutant compared to the WT, while four endoglucanases were increased in the extracellular medium of the Δ3238 mutant. Six endoglucanases detected in the extracellular medium of the Δ3238 mutant were not found in the extracellular medium of the WT strain (Supplementary Table 1). Determination of the active endoglucanases in the native gel showed that the outer membrane and the extracellular space in the WT showed higher endoglucanase activities than the Δ3238 mutant (Supplementary Figure 6). This suggests that loss of *chu_3238* caused most of the endoglucanases to fail to anchor to the outer membrane with the result that they are released, while no increased endoglucanase activity was determined in the extracellular medium of the Δ3238 mutant, reflecting abnormal forms of endoglucanases in the Δ3238 extracellular medium. However, approximately 80% endoglucanase activity was observed in the lysed cells (excluding the extracellular medium) of the Δ3238 mutant when compared to lysed cells of the WT. It is possible that the expression of the unpredicted intracellular endoglucanases was increased in the Δ3238 mutant; therefore, minor changes in endoglucanase activity were observed in the lysed cells of the Δ3238 mutant. The decreased outer membrane proteins may be the main reason for the decreased cellulose-binding ability of the Δ3238 mutant (Figure 6). Overall, the decreased outer membrane proteins, including cellulases, may be the main cause of cellulolytic defects in the Δ3238 mutant because these proteins are involved in cellulose degradation and binding (Ji et al., 2012, 2014; Wang et al., 2017, 2019; Zhao et al., 2020).

In *P. gingivalis*, CTD proteins are anchored to the cell surface *via* a covalent linkage to an A-LPS on the outer membrane (Gorasia et al., 2015). PorV can also act as LptO to function in the *O*-deacylation of A-LPS (Chen et al., 2011). However, it was not clear whether the CTD proteins in *C. hutchinsonii* were modified by A-LPS. Defects in the Congo red staining of the cell surface and the absence of the LPS band as observed in the Tris-Tricine-SDS-PAGE indicated that the Δ3238 mutant had an incomplete LPS (Figures 7A,B). The destruction of LPS integrity may be the reason for the disrupted cell integrity, increased outer membrane permeability, and increased cell sensitivity to toxic reagents in the Δ3238 mutant (Figure 7C). The relationship between CHU_3238 and LPS structure and whether the cargo proteins of the T9SS are anchored to the cell surface by LPS modification in *C. hutchinsonii* need to be further studied. Moreover, PorV, a shuttle protein, interacts with other T9SS components to transport cargo proteins (Glew et al., 2017; Lauber et al., 2018). Through the use of an anti-FLAG antibody, we identified CHU_3238 in the outer membrane and total membrane of the 3238-FLAG strain (Figure 8A), suggesting that it was localized in the outer membrane. Proteins interacting with CHU_3238 were identified using co-immunoprecipitation in the 3238-FLAG strain. These co-immunoprecipitated proteins (marked with arrowheads) were identified as homologous to a T9SS component, SprA (CHU_0029), and a TonB-dependent receptor (CHU_1606) (Figure 8B and Table 3). The TonB-dependent receptor is responsible for Fe transport and has a Plug domain that acts as the channel gate. CHU_1606 showed 32.8% sequence identity in amino acid with PG0534 (PorF, a TonB-dependent receptor), a T9SS component in *P. gingivalis*. PG0534, an outer membrane protein, was required for normal secretion of gingipains in *P. gingivalis* (Saiki and Konishi, 2010; Lasica et al., 2017; Gorasia et al., 2020a). While how PorF associates with other T9SS component is unknown and PG0534 did not have the Plug domain, the relationship of PorV and PorF requires to be further explored. It was reported that the translocon SprA forms a complex with PorV and the Plug protein as the transport channel of the T9SS in *F. johnsoniae*. *C. hutchinsonii* may form the same complex to transport the T9SS cargo proteins to the outer membrane.

PorV is the most abundant T9SS component (Gorasia et al., 2020a) and has multiple functions, suggesting its important role in the T9SS. Previous studies have reported that CTD proteins in *porV* mutants have uncleaved CTDs, and their secretion is blocked in the periplasm (Chen et al., 2011; Glew et al., 2017). In *F. johnsoniae*, PorU plays no role in the T9SS, and loss of PorV results in disabled substrate secretion (Kharade and McBride, 2015). Moreover, gliding motility was not affected in the *porV* deletion mutants of *F*. *johnsoniae* and *Flavobacterium columnare*. In *C. hutchinsonii*, deletion of CHU_3238 caused gliding defects and resulted in secreted proteins being released into the extracellular medium, thus showing the functional difference of PorV in *C. hutchinsonii* from other *Bacteroidetes*. We also detected that CHU_3238 affected the structure of LPS and may form a complex with a SprA homolog and a Plug protein, which is consistent with previous reports on the function of PorV. The functional diversity of CHU_3238 and its difference from other PorV proteins indicate that the T9SS is involved in many physiological processes in *C. hutchinsonii*. CHU_3238 as PorV is worth exploring to help understand the functions of the T9SS in *C. hutchinsonii* and to further investigate cellulose degradation, gliding, and ion assimilation mechanisms.

## Data Availability Statement

The raw data supporting the conclusions of this article will be made available by the authors, without undue reservation.

## Author Contributions

DZ, WS, SW, and XL conceived and designed the research. DZ and WS conducted the experiments. SW, WZ, and YZ performed statistical analyses. DZ, WS, and XL wrote the manuscript. All authors contributed to the article and approved the submitted version.

## Conflict of Interest

The authors declare that the research was conducted in the absence of any commercial or financial relationships that could be construed as a potential conflict of interest.

## Publisher’s Note

All claims expressed in this article are solely those of the authors and do not necessarily represent those of their affiliated organizations, or those of the publisher, the editors and the reviewers. Any product that may be evaluated in this article, or claim that may be made by its manufacturer, is not guaranteed or endorsed by the publisher.
