# Supplementary material for: Identification of the Type IX Secretion System Component, PorV (CHU_3238), Involved in Secretion and Localization of Proteins in Cytophaga hutchinsonii
Source: Front Microbiol. 2021 Oct 20;12:742673. doi: 10.3389/fmicb.2021.742673 (PMC8564354; doi:10.3389/fmicb.2021.742673)
Supplement: Supplementary file 5 [file Data_Sheet_5.PDF]

Supplementary Material

1 Supplementary Figures and Tables

1.1 Supplementary Figures

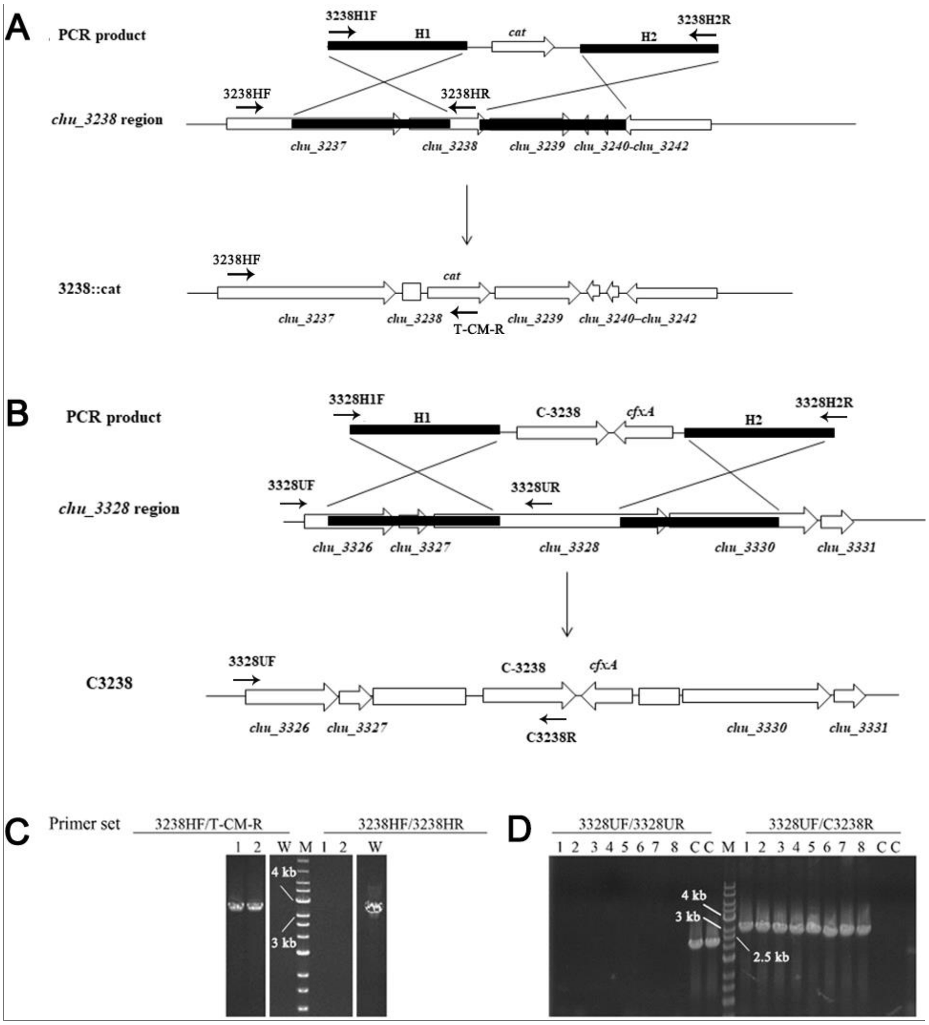

Supplementary Figure 1. Deletion and complementation of *chu_3238*. (A) Schematic representation of the deletion of *chu_3238*. The arrowheads indicated approximate locations and direction of primers (B) PCR verification of the Δ3238 mutant with two set of primers 3238HF/T-CM-R (about 4 kb), and 3238HF/3238HR (about 3.7 kb). 1-7, seven transformants of the Δ3238 mutant; W, the wild-type strain; M, DNA marker. (C) PCR verification of the complemented strain C3238 with two sets of primers 3328UF/3328UR (about 2.2 kb) and 3328UF/C3238R (about 3.7 kb). 1-8, eight transformants of the complemented strain C3238; C, the Δ3238 mutant; M, DNA marker.

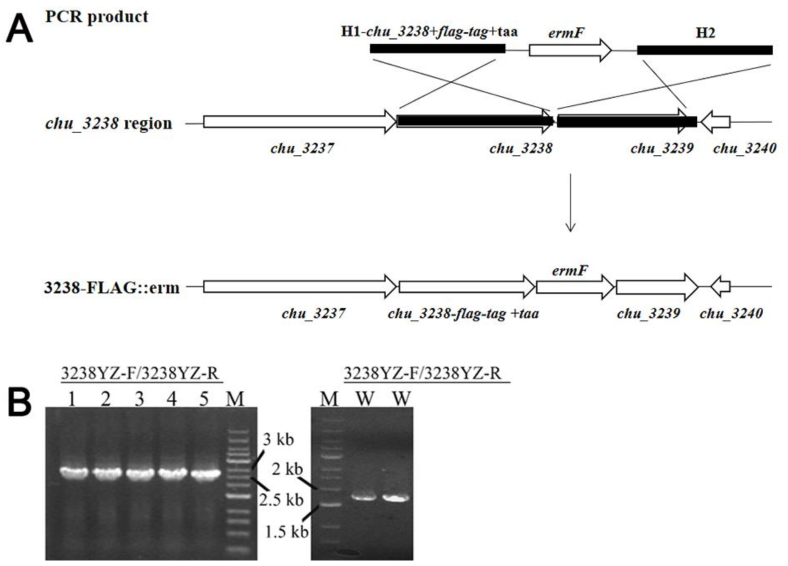

Supplementary Figure 2. Construction of the 3238-FLAG strain. (A) Schematic representation of inserting the FLAG-tag at the C-terminal of chu_3238. (B) PCR verification of the Δ3238 mutant with primers 3238YZ-F/3238YZ-R. 1-5, five transformants of the Δ3238 mutant; W, the wild-type strain; M, DNA marker.

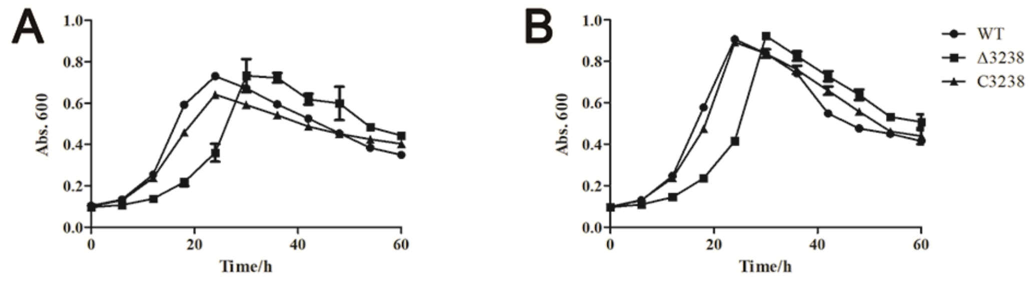

Supplementary Figure 3. Growth curves of the wild type (WT), the Δ3238 mutant and the complemented strain C3238 in PYS medium with 0.4% (w/v) glucose (A) or 0.4% (w/v) cellobiose (B) as the sole carbon source. Cells were grown at 30°C

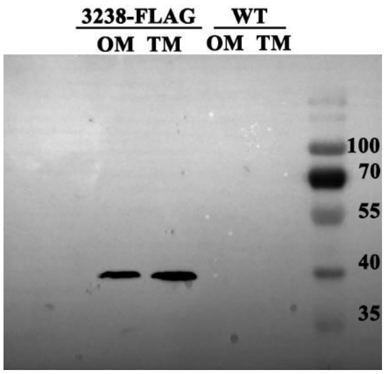

Supplementary Figure 4. Full size of PVDF membrane of Western blot in Figure 8A.

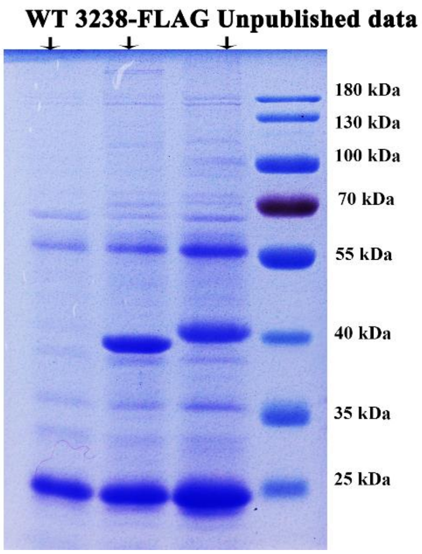

Supplementary Figure 5. Full size of SDS-PAGE gel in Figure 8B.

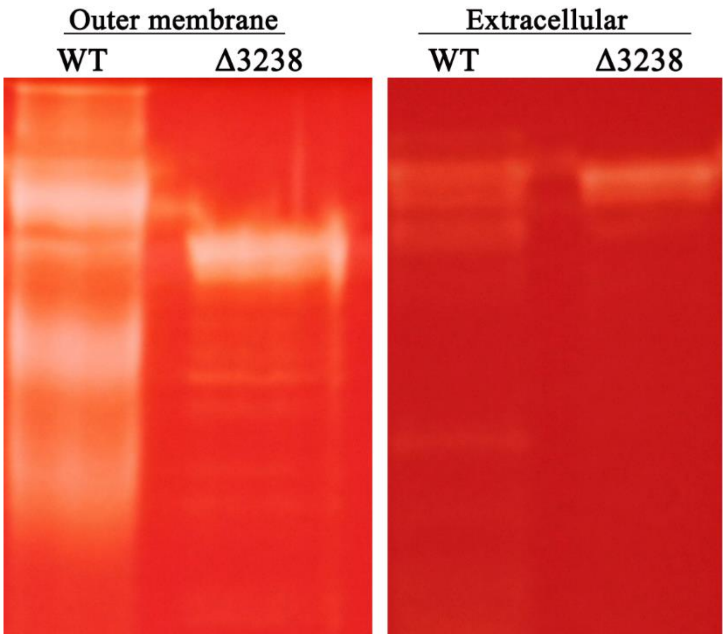

Supplementary Figure 6. The active endoglucanases of the outer membrane and the extracellular space in the wild type (WT) and the Δ3238 mutant in the native gel.
