# Supplementary material for: Identification of the Type IX Secretion System Component, PorV (CHU_3238), Involved in Secretion and Localization of Proteins in Cytophaga hutchinsonii
Source: Front Microbiol. 2021 Oct 20;12:742673. doi: 10.3389/fmicb.2021.742673 (PMC8564354; doi:10.3389/fmicb.2021.742673)
Supplement: Supplementary file 6 [file Table_1.DOCX]

Supplementary Material

## Supplementary Table

**Supplementary Table 1** Distribution of predicted endoglucanases by proteomic analysis ^α^

|  |  |  |  | Spectral scores | | | | |
| --- | --- | --- | --- | --- | --- | --- | --- | --- |
|  | CHU. no | Location | CTD | WT-OM | Δ3238-OM | WT-EX | | Δ3238-EX |
| Cel5A | CHU_1107 | CM | YES | 1826 | 167 | 172 | | 667 |
| Cel5B | CHU_2103 | OM | NO | 187 | 198 | 34 | | 455 |
| Cel5C | CHU_1727 | EX | NO | 158 | 30 | - | | 295 |
| Cel5D | CHU_1842 | CM | NO | 228 | - | - | | - |
| Cel5E | CHU_3727 | EX | YES | 506 | - | - | | 102 |
|  | CHU_3441 | OM | YES | 191 | - | - | | - |
|  | CHU_3440 | OM | YES | - | - | - | | - |
|  | CHU_1240 | EX | YES | 39 | - | - | | - |
|  | CHU_1075 | EX | YES | 2803 | 274 | 61 | | 1735 |
|  | CHU_2149 | OM | YES | - | - | | - | - |
|  | CHU_2852 | EX | YES | - | - | | - | - |
| Cel9E | CHU_0778 | EX | NO | - | - | | - | - |
| Cel | CHU_0961 | EX | YES | 458 | - | | - | 86 |
| Cel9C | CHU_1280 | EX, P | NO | 1013 | 341 | | 139 | 1337 |
| Cel9B | CHU_1335 | EX | YES | 195 | - | | - | 327 |
| Cel9A | CHU_1336 | EX | YES | 845 | - | | - | 573 |
| Cel9D | CHU_1655 | CM, P, EX | YES | 242 | - | | - | 47 |
| Cel9F | CHU_2235 | EX | NO | - | - | | - | - |

^α^ The location is predicted with pSORTb 3.0. CTD, conserved in the proteins that were identified and translocated by the T9SS. “YES” is indicated with CTD in the protein, and “No” is indicated without CTD in the protein. WT-OM, the spectral scores of proteins in the outer membrane of the wild type. Δ3238-OM, the spectral scores of proteins in the outer membrane of the Δ3238 mutant. WT-EX, the spectral scores of proteins in the extracellular medium of the wild type. Δ3238-EX, the spectral scores of proteins in the extracellular medium of the Δ3238 mutant. CM, cytoplasmic membrane. OM, outer membrane. EX, extracellular. P, periplasm.
